# Factors That Influence Conversion to Resectability and Survival After Resection of Metastases in RAS WT Metastatic Colorectal Cancer (mCRC): Analysis of FIRE-3- AIOKRK0306

**DOI:** 10.1245/s10434-020-08219-w

**Published:** 2020-03-14

**Authors:** Dominik Paul Modest, Volker Heinemann, Gunnar Folprecht, Timm Denecke, Johann Pratschke, Hauke Lang, Marc Bemelmans, Thomas Becker, Markus Rentsch, Daniel Seehofer, Christiane J. Bruns, Bernhard Gebauer, Swantje Held, Arndt Stahler, Kathrin Heinrich, Jobst C. von Einem, Sebastian Stintzing, Ulf P. Neumann, Ingrid Ricard

**Affiliations:** 1 Department of Hematology, Oncology and Tumor Immunology, CVK, Charité Universitätsmedizin Berlin, Berlin, Germany; 2 Medical Department III and Comprehensive Cancer Center, Hospital of the University, Ludwig-Maximilian-University (LMU), Munich, Germany; 3 University Cancer Center/Medical Department I, University Hospital Carl Gustav Carus, Dresden, Germany; 4 Institute of Radiology, Charité, Berlin, Germany; 5 General, Visceral, and Transplantation Surgery, Charité - Universitätsmedizin Berlin, Berlin, Germany; 6 Klinik für Allgemein-, Viszeral- und Transplantationschirurgie, Universitätsmedizin Mainz, Mainz, Germany; 7 Department of Surgery, Maastricht University Medical Centre, Maastricht, The Netherlands; 8 Klinik für Allgemeine-, Viszeral-, Thorax-, Transplantations- und Kinderchirurgie, Universitätsklinikum Schleswig-Holstein, Campus Kiel, Kiel, Germany; 9 Department of General, Visceral, Transplantation, Vascular and Thoracic Surgery, Hospital of the University of Munich, Munich, Germany; 10 Klinik und Poliklinik für Visceral-, Transplantations-, Thorax- und Gefäßchirurgie Universitätsklinikum Leipzig, Leipzig, Germany; 11 Klinik und Poliklinik für Allgemein-, Viszeral- und Tumorchirurgie, Universitätsklinikum Köln, Cologne, Germany; 12 ClinAssess GmbH, Leverkusen, Germany; 13 Department of General, Visceral and Transplantation Surgery, University Hospital Aachen, Aachen, Germany; 14 Comprehensive Cancer Center, Hospital of the University, Ludwig-Maximilian-University (LMU), Munich, Germany

## Abstract

**Background:**

Tumor assessments after first-line therapy of *RAS* wild-type mCRC with cetuximab (cet) versus bevacizumab (bev) in combination with FOLFIRI were evaluated for factors influencing resectability, conversion to resectability, and survival after best response.

**Methods:**

Conversion to resectability was defined as conversion of initially unresectable to resectable disease at best response as determined by retrospective assessment. Univariate and multivariate logistic models were fitted with resectability at best response as response variable. A Cox model comparing the survival from best response was used to measure the influence of treatment, resectability at best response, and resection. Interaction of resection and treatment arm on survival was tested by likelihood ratio test.

**Results:**

Overall, 270 patients were evaluable (127 cet-arm, 143 bev-arm). Lung metastases (odds ratio [OR] 0.35, 95% confidence response [CI] 0.19–0.63), *BRAF* mutation (OR 0.33, 95% CI 0.12–0.82), and elevated alkaline phosphatase (OR 0.42, 95% CI 0.18–0.9) before randomization were associated with less chance of successful conversion and were integrated into a nomogram. Early tumor shrinkage (OR 1.86, 95% CI 1.06–3.3; *p* 0.034) and depth of response (OR 1.02, 95% CI 1.01–1.03; *p* < 0.001) were associated with successful conversion therapy. Resection of metastases improved post-best-response survival (hazard ratio 0.53, 95% CI 0.29–0.97; *p* = 0.039), predominantely in cet-treated patients (interaction test, *p* = 0.02).

**Conclusions:**

Conversion to resectability is significantly associated with baseline characteristics that can be used in a nomogram to predict conversion. Moreover, early efficacy parameters (ETS and DpR) are associated with successful conversion therapy. In FIRE-3, resection of metastases was associated with improved post-best response survival, this effect originated predominantly from the cetuximab-based study arm.

**Electronic supplementary material:**

The online version of this article (10.1245/s10434-020-08219-w) contains supplementary material, which is available to authorized users.

Multidisciplinary treatment of metastatic colorectal cancer (mCRC), including surgery and/or ablative techniques, improves the outcome of patients and provides a chance for long-term survival and eventually cure.^1^^–^3 The criteria to select patients for resection are not yet standardized and may differ from one unit to another, as well as between surgeons and medical oncologists.^4^^–^6 Accordingly, the current ESMO guideline recommends secondary interventions in patients with oligo-metastatic disease—defined rather vaguely as limited (in terms of lesions and involved organs) disease spread.^2^

Consequently, the concept of oligometastatic disease allows for local therapy in patients with mCRC with the disease not necessarily limited to one organ or an exact number of lesions.^2^ Therefore, the selection of candidates for multidisciplinary therapy has become less precise, more individual, and more challenging. Decisions rely on the experience and technical abilities of individual multidisciplinary teams and their preferred algorithms and tools (in particular concerning ablative techniques).^2^ Given that no exact (positive or negative) precondition for potential resectability can be defined, the repeated case-by-case discussion in multidisciplinary conferences, including experienced specialists, is a key factor for optimal management of mCRC.^2^,^7^

The patient population of the FIRE-3 trial was recruited in more than 100 centres across Germany and Austria. Inclusion into the trial required measurable disease, but selection according to spread of disease was not performed. Accordingly, the FIRE-3 cohort may be considered an average, mixed population concerning the pattern of lesions.^8^^–^12 This trial population was retrospectively evaluated for surgical and/or multidisciplinary treatment options based on a central review of imaging. This exploratory analysis suggested that locoregional interventional treatment might have been possible in markedly more patients than was actually performed.^13^ Furthermore, the central review provided scores of the presented images for technical difficulty and prognostic impact of a potential intervention.

In the present investigation, we used the FIRE-3 database on resectability to identify factors that correlated with resectability before and after treatment. In this context, we specifically searched for factors that may help to predict successful conversion to resectability (not resectable before, but after systemic therapy). In addition, we analysed the outcome of resected patients according to the systemic treatment used (cetuximab vs. bevacizumab) in the trial. This investigation represents a large data base on resection of metastases in mCRC relying on intensive characterisation of images. Because this analysis is exploratory, the results should be interpreted as such.

## Methods

### Patients

Patients who were eligible for this investigation were treated in FIRE-3 and received first-line treatment for mCRC with FOLFIRI in combination with either cetuximab or bevacizumab. The initial protocol recruited patients irrespective of molecular information. After an amendment, the study was restricted to patients with *KRAS* exon 2 wild-type tumors. For the present investigation, we only analysed patients of the *RAS* wild-type cohort, which was identified retrospectively. Patients analysed in this manuscript also had a central assessment for resectability.^13^ Central evaluation of tumor response, early tumor shrinkage (ETS), and depth of response (DpR) also were available.^14^ For details concerning treatment and outcomes of the study, please refer to previous publications.^9^^,^^14^^,^^15^ The trial is registered with ClinicalTrials.gov-identifier NCT0043392.

### Central Evaluation of Resectability

The full details and primary results of central assessment for resectability were published previously.^13^ Briefly, baseline and best response images were evaluated for interventional options and resectability was defined, if at least 50% of the reviewers opted for intervention (8 surgeons, 3 medical oncologists). Furthermore, for each tumour assessment an evaluation for technical difficulty (1 = very easy to 10 = impossible) and anticipated clinical benefit (1 = great benefit to 10 = no benefit) was done on a visual analogue scale.^13^

### Definition of Conversion to Resectability

Conversion to resectable disease was assumed in patients that were considered unresectable at baseline (< 50% votes for intervention) and were evaluated as resectable according to the best response image (> 50% votes for intervention).

### Statistics

The influence of baseline information on resectability of metastases and first-line treatment was assessed with Fisher’s exact tests for categorical variables and with Kruskal-Wallis rank sum tests for continuous ones. If dependence was detected, further tests were performed in order to determine if resectability depends on treatment. It was also tested among patients who were assessed as resectable at best response, if baseline characteristics of patients who were resected differ from the ones of patients who were not.

Conversion to resectability was defined as resectable disease at best response and evaluated in patients with unresectable disease before randomization. Univariate and multivariate logistic models were fitted with resectability at best response as response variable. A reduced model was built by combining bootstrap resampling with backward elimination based on Wald chi-square test of individual factors. The significance level for staying in a model was set to 0.05. Five hundred bootstrap samples were generated, and variables selected in at least 60% of the bootstrap samples were included in the final model.^16^ A nomogram based on the resulting model was constructed to predict successful conversion therapy (Fig. 4). Resampling validation was used to assess the performance of the reduced model. The bias-corrected AUC, the intercept and slope of the overall logistic calibration equation, the maximum absolute difference in predicted, and calibrated probabilities (*E*_max_) were computed.

Cox models comparing survival from best response were fitted to measure the influence of treatment, resectability at best response and resection. As all resections naturally took place after best response this variable was included as a time-dependent variable. Models composed of different combinations of these three explanatory variables main, simple, and/or first-order interaction effects were fitted. Akaike information criterion (AIC) was used to select the best model. Interaction of resection and treatment arm on survival was tested by likelihood ratio test.

All tests were two-sided with a significance level of 0.05. Statistical analyses were performed using R (version 3.4.1) and more particularly the packages survival, multcomp, forestplot, and rms.

### Funding

The sponsor of the FIRE-3 trial is the university hospital of the LMU Munich. The trial received financial support from Merck and Pfizer (no grant numbers). These sources had no role in the decision to submit or publish the data. DPM and VH had the final responsibility to submit and publish the data.

## Results

### Patients Included in the Analysis

**Fig. 1:**
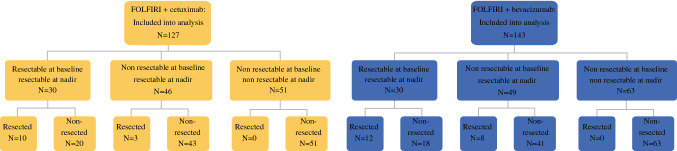
Study arms, resectability, and performed resections (RAS wild-type population). *One patient in the bevacizumab arm was resectable at baseline and became unresectable at best response (nadir) and was consecutively not resected

**Fig. 2:**
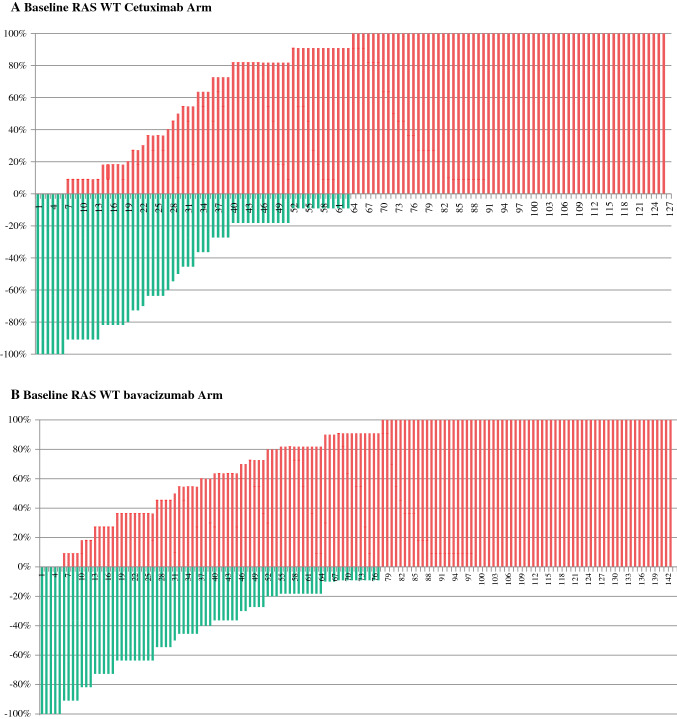

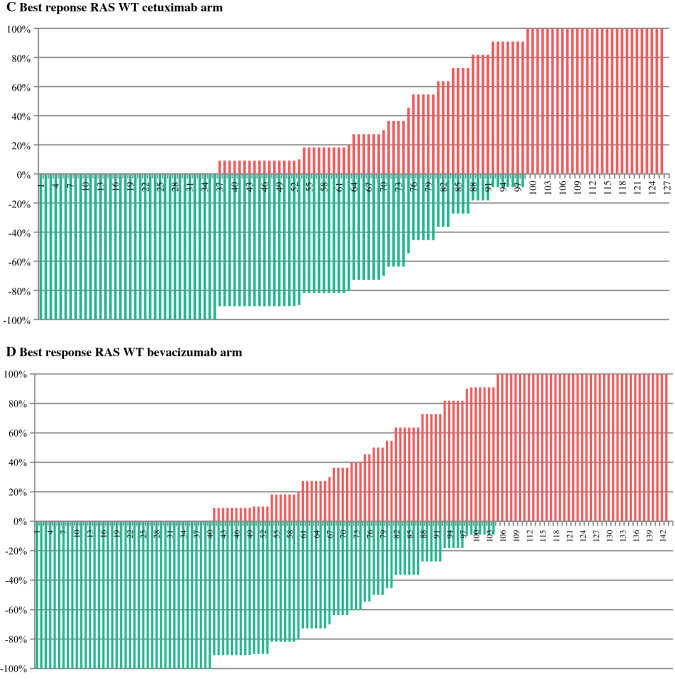
Resectability review of the RAS wild-type subset. For baseline assessments, original scores were simplified to “yes” (=R0 resection with or without perioperative therapy only limited to the abdomen or not) and “no” (=conversion therapy or not resectable). For the best response assessment original scores were simplified to “yes” (=R0 resection with or without ablative modality limited to the abdomen or not) and “no” (=not resectable) 13

Of the full FIRE-3 population, 270 patients with *RAS* wild-type mCRC were evaluable for central review of resectability and were included into this analysis. A consort diagram, illustrating the populations of the study can be found as supplementary Fig. 1. The subgroups according to the central review and performed resections are summarized in Fig. 1. The original perspectives (simplified as yes/no answer) of the central review on the *RAS* wild-type population are displayed as Fig. 2a–d.

Characteristics of patients and tumors according to review assessment (resectable or not resectable at best response) and reported outcome in the study (resected or not resected) are summarized in Table 1. In the univariate analysis, several baseline variables, such as age at randomization (*p* = 0.0043), number of metastatic sites (*p* = 0.0007), presence of lung metastasis (*p* = 0.00045), presence of other metastasis (*p* = 0.0057), liver limited disease (*p* = 0.0054), and alkaline phosphatase (*p* = 0.043), appeared to have a significant positive/negative influence on outcome after first-line treatment, i.e., resectability and resection (Supplementary Figs. 2A–E). Few contrasts of interest reached statistical significance in a linear regression model (Table 2).

**Table 1:** Characteristics of patients and tumors included in the study

	FOLFIRI + bevacizumab			FOLFIRI + cetuximab			*p* value
	Nonresectable/nonresected (*n* = 64)	Resectable/nonresected (*n* = 59)	Resectable and resected (*n* = 20)	Nonresectable/nonresected (*n* = 51)	Resectable/nonresected (*n* = 63)	Resectable and resected (*n* = 13)	
Age at randomization (years)	62.1 (10)	60.4 (8.7)	65.9 (6.2)	66.3 (6.9)	63.3 (8.4)	61.2 (8.3)	0.0043
Number of metastatic sites	2 (1)	1.7 (0.8)	1.6 (0.8)	2.4 (1.1)	1.7 (0.9)	1.5 (0.7)	0.0007
Center type							0.44
University hospital	14 (21.9)	6 (10.2)	5 (25)	8 (15.7)	12 (19)	3 (23.1)	
Other hospital or practice	50 (78.1)	53 (89.8)	15 (75)	43 (84.3)	51 (81)	10 (76.9)	
Sex							0.74
Female	23 (35.9)	20 (33.9)	4 (20)	16 (31.4)	17 (27)	3 (23.1)	
ECOG							0.78
0	31 (48.4)	32 (54.2)	12 (60)	24 (47.1)	37 (58.7)	7 (53.8)	
1–2	33 (51.6)	27 (45.8)	8 (40)	27 (52.9)	26 (41.3)	6 (46.2)	
Tumour side:							0.25
Left-sided	46 (71.9)	48 (81.4)	19 (95)	37 (72.5)	50 (79.4)	12 (92.3)	
Right-sided	17 (26.6)	10 (16.9)	1 (5)	12 (23.5)	12 (19)	1 (7.7)	
Missing	1 (1.6)	1 (1.7)	0 (0)	2 (3.9)	1 (1.6)	0 (0)	
Metastatic sites							
Liver	51 (79.7)	51 (86.4)	20 (100)	45 (88.2)	55 (87.3)	13 (100)	0.17
Lung	30 (46.9)	15 (25.4)	4 (20)	26 (51)	21 (33.3)	0 (0)	0.00045
Lymph nodes	25 (39.1)	17 (28.8)	6 (30)	22 (43.1)	18 (28.6)	4 (30.8)	0.51
Peritoneum	6 (9.4)	5 (8.5)	1 (5)	7 (13.7)	3 (4.8)	0 (0)	0.57
Other	19 (29.7)	14 (23.7)	0 (0)	20 (39.2)	12 (19)	2 (15.4)	0.0057
Liver limited disease	17 (26.6)	21 (35.6)	12 (60)	11 (21.6)	26 (41.3)	8 (61.5)	0.0054
Lung limited disease	4 (6.2)	1 (1.7)	0 (0)	2 (3.9)	2 (3.2)	0 (0)	0.81
Prior radiotherapy							0.23
Yes	9 (14.1)	3 (5.1)	5 (25)	5 (9.8)	8 (12.7)	1 (7.7)	
No	55 (85.9)	56 (94.9)	15 75)	46 (90.2)	54 (85.7)	12 (92.3)	
Missing	0 (0)	0 (0)	0 (0)	0 (0)	1 (1.6)	0 (0)	
Prior adjuvant treatment							0.18
Yes	10 (15.6)	5 (8.5)	6 (30)	11 (21.6)	12 (19)	1 (7.7)	
No	54 (84.4)	54 (91.5)	14 (70)	40 78.4)	50 (79.4)	12 (92.3)	
Missing	0 (0)	0 (0)	0 (0)	0 (0)	1 (1.6)	0 (0)	
Leucocytes							0.24
< 8000 /nL	38 59.4)	32 54.2)	16 80)	26 (51)	33 (52.4)	7 (69.2)	
≥ 8000 /nL	26 (40.6)	27 (45.8)	4 (20)	25 (49)	30 (47.6)	4 (30.8)	
Alkalic phosphatase							0.043
< 300 U/L	49 (76.6)	54 (91.5)	18 (90)	41 (80.4)	58 (92.1)	13 (100)	
≥ 300 U/L	15 (23.4)	5 (8.5)	2 (10)	10 (19.6)	5 (7.9)	0 (0)	
Development of metastases							0.75
Synchronous metastatic disease	54 (84.4)	45 (76.3)	15 (75)	41 (80.4)	46 (73)	11 (84.6)	
Metachronous metastatic disease	10 (15.6)	13 (23.7)	4 (25)	10 (19.6)	16 (25.4)	2 (15.4)	
Missing	0 (0)	0 (0)	0 (0)	0 (0)	1 (1.6)	0 (0)	
BRAF							0.18
Mutant	11 (17.2)	(6.8)	0 (0)	5 (15.7)	6 (9.5)	1 (7.7)	
Wild-type	53 (82.8)	55 (93.2)	20 (100)	41 (80.4)	57 (90.5)	12 92.3)	
Missing	0 (0)	0 (0)	0 (0)	2 (3.9)	0 (0)	0 (0)	

Representation of mean (standard deviation) when the variable is continuous, number (%) when the variable is categorical. Resectability was assessed at best response. *p* value are drawn from Fisher exact tests when the variable is categorical and from Kruskal-Wallis rank sum test when it is continuous (age at randomization and number of metastatic sites)

**Table 2:** Characteristics of patients and tumors included in the study—focus on some particular comparisons of interest

	Difference between the two treatment arms among (cetuximab was taken as reference)						Difference between resected and nonresected among patients who were assessed as resectable at best response (resected group is the reference group)					
	Nonresectable at best response/nonresected patients		Resectable at best response/nonresected patients		Resectable at best response/resected patients		All resectable patients		Bevacizumab patients		Cetuximab patients	
	Estimate (95% CI)	*p*	Estimate (95% CI)	*p*	Estimate (95% CI)	*p*	Estimate (95% CI)	*p*	Estimate (95% CI)	*p*	Estimate (95% CI)	*p*
Age at randomization (yr)	− 4.23 (− 7.37 to − 1.09)	0.042	− 2.98 (− 6.01–0.05)	0.22	4.67 (− 1.29–10.63)	0.37	− 3.41 (− 10.09–3.28)	0.410	− 5.53 (− 9.85 to − 1.2)	0.062	2.12 (− 2.98–7.21)	0.410
Number of metastatic sites	0.87 (0.74–1.02)	0.56	1.00 (0.84–1.19)	0.99	1.06 (0.73–1.54)	0.99	1.32 (0.87–2)	0.76	1.12 (0.86–1.45)	0.99	1.18 (0.86–1.63)	0.90
Lung metastasis	1.18 (0.53–2.63)	0.77	1.46 (0.62–3.49)	0.77	0.00 (0–2.24)	0.54	0.33 (0.08–1.04)	0.23	0.74 (0.15–2.8)	0.77	0.00 (0–0.74)	0.089
Other metastasis	1.52 (0.65–3.57)	0.97	0.76 (0.29–1.97)	> 0.99	Inf* (0.3 − Inf)	0.59	0.24 (0.03–1.05)	0.22	0 (0–0.77)	0.096	0.78 (0.07–4.32)	> 0.99
Liver limited disease	0.76 (0.29–1.96)	> 0.99	1.27 (0.57–2.82)	> 0.99	1.06 (0.21–5.78)	> 0.99	2.44 (1.04–5.89)	0.18	2.68 (0.85–8.89)	0.35	2.25 (0.57–9.8)	0.88
Alkaline phosphatase < 300 U/L	0.80 (0.29–2.14)	> 0.99	0.93 (0.2–4.29)	> 0.99	0.00 (0–8.22)	> 0.99	0.72 (0.07–3.66)	> 0.99	1.20 (0.11–8.13)	> 0.99	0.00 (0–5.48)	> 0.99

Estimates consist in odds ratio when the variable is binary. *p* values are then drawn from Fisher exact tests. A linear regression model was fitted with age at randomization as response variable. Estimates displayed are pairwise differences. As the number of metastatic sites are integers lying between 1 and 5 relative risks drawn from a Poisson regression are displayed. Hommel procedure was used to adjust the *p* values for multiplicity

*Infinite odds ratio is due to a null cell frequency (no patient with other metastasis, and treated with bevacizumab was assessed as resectable at best response and resected)

*CI* confidence interval, *p* adjusted *p* value, *Inf* infinity

### Baseline Factors and Efficacy Parameters Correlating with Conversion to Resectability

**Fig. 3:**
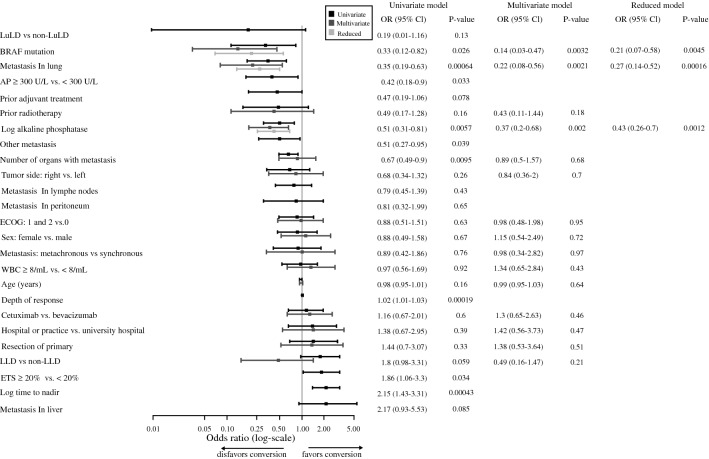
Effect of potential predictors on the probability of becoming resectable at best response. Representation of unadjusted odds ratios (logistic univariate models), odds ratios adjusted for other predictors (logistic multivariate model) and odds ratios of the reduced model. Only patients whose tumor lesions were assessed as non-resectable at baseline were kept in the analyses. The response variable is binary and equals 1 if the patient status became resectable at best response and zero otherwise. Post-baseline variables (log time to no best response, ETS, Depth of response) were excluded from the multivariate analyses as well as some variables due to correlation/colinearity (prior adjuvant treatment was removed, because it is highly correlated with metastasis type, some variables in link with the presence of metastasis in an organ and the number of organs with metastasis, the dichotomous version of alkaline phosphatase as the log-transformed version was included in the analysis). *LLD* liver limited disease; *LuLD* lung limited disease; *ETS* early tumor shrinkage; *AP* alkaline phosphatase; *WBC* white blood cell count. In the multivariate analyses, LLD patients seem to have less chance to be resectable at best response than non-LDD patients. The reason for this is the presence of the variable “lung metastasis” and “Number of organs with metastasis” in the model. Once these two variables are removed from the analyses, LLD OR is greater than 1 (but *p* value is still > 0.05)

As shown in Fig. 3, the presence of lung metastasis (OR 0.35, 95% CI 0.19–0.63; *p* < 0.001), *BRAF* mutation (OR 0.33, 95% CI 0.12–0.82; *p* = 0.026), and alkaline phosphatase > 300 U/L (OR 0.42, 95% CI 0.18–0.9; *p* = 0.033) before randomization were significantly associated with less chance of conversion to resectability. These effects remain statistically significant once adjusted for other baseline characteristics. Notably, pretreatment (with adjuvant chemotherapy) also showed a trend to negatively correlate with resectability. Favorable treatment associated markers, such as early tumor shrinkage (ETS, ≥ 20% reduction of tumor diameter at 6 weeks) and depth of response (DpR, greatest reduction of tumor diameter) were significantly associated with conversion to resectability (ETS: OR 1.86, 95% CI 1.06–3.3, *p* = 0.034; DpR: OR 1.02, 95% CI 1.01–1.03, *p* < 0.001).

Presence of lung metastasis, BRAF mutation, and log-transformed alkaline phosphatase were the variables selected as predictors in more than 60% (even 80%) of the bootstrap samples. Moreover the corresponding log OR estimates were of same sign for all bootstrap samples, indicating good model stability. Resampling validation of the model with these three variables as predictor leads to AUC = 0.708 and *E*_max_ = 0.013. The intercept and slope of the overall logistic calibration equation were estimated to − 0.010 and 0.952, respectively, so that the probability of successful conversion therapy in RAS wild type mCRC can be predicted by

$$\begin{aligned} & [1 + { \exp }( - ( - 0.010 + 0.952 \times (4.659 - 1.305 \, \left( {{\text{if}}\,{\text{lung}}\,{\text{metastasis}}} \right) \\ & \quad - 1.543\left( {{\text{if}}\,{\text{BRAF}}\,{\text{mutation}}} \right) - 0.844 \times ({ \log }\,{\text{alkaline}}\,{\text{phosphatase}}\,{\text{at}}\,{\text{baseline}}))]^{ - 1} . \\ \end{aligned}$$

**Fig. 4:**
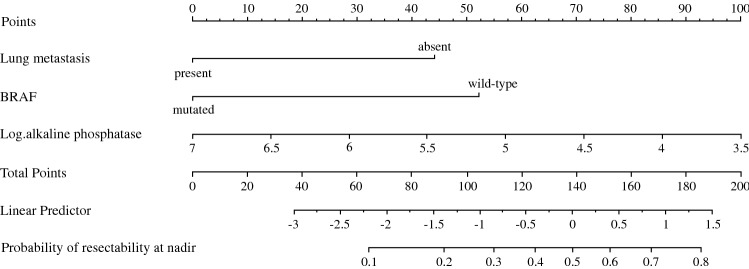
Nomogram for the prediction of successful conversion therapy. To get a patient prediction of resectability at nadir: Step 1, compute the number of points corresponding to the patient by drawing vertical lines from patient lung metastasis status, BRAF status and log-transformed alkalic phosphatase value to the “Points” scale. A patient without any lung metastasis, a wild-type BRAF tumor and log transformed baseline alkalic phosphatase of 4 would get a total number of points of 44 + 52 + 86 = 182. Step 2, report this value on “Total Points” scale and draw a vertical line passing through the value 182. The intersection of this vertical line with “Probability of resectability at nadir” scale gives the patient probability of resectability at nadir

A nomogram also can be used to predict successful conversion therapy in RAS wild-type mCRC (Fig. 4).

### Post-best Response Survival Model

**Fig. 5:**
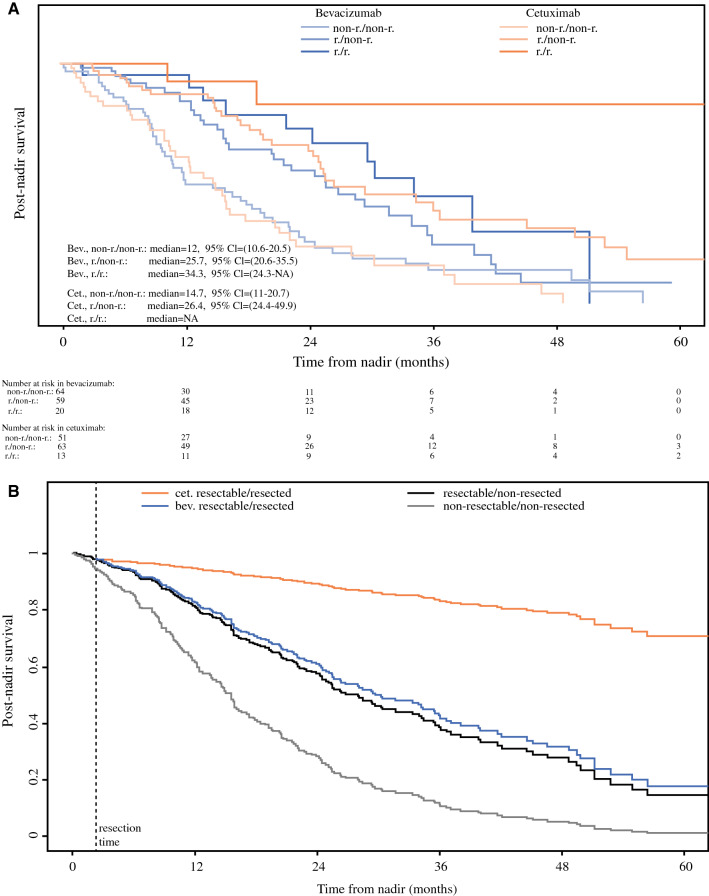
**a** Kaplan–Meier plot representing post-best response survival for each treatment—resectability at best response—resected status group. **b** Fitted post best response survival based on Model with smallest AIC according to resectability at best response, resection, and first-line treatment

All 270 patients were at risk until at least best response. Resection of metastases significantly improved post-best response survival (HR 0.53, 95% CI 0.29–0.97, *p* = 0.04). This observation was specifically made in cet-treated patients, but not in bev-treated patients (HR (cet) 0.17, 95% CI 0.04–0.69, *p* = 0.01; HR (bev) 0.89, 95% CI 0.47–1.69, *p* = 0.73; interaction test *p* = 0.02). The model with the smallest AIC is the model containing resectability at best response as main effect and the interaction effect of resection with treatment. Kaplan-Meier post best response survival curves of each treatment / resectability / resected status subgroups are represented in Fig. 5a and fitted curves of the best model in Fig. 5b.

The inclusion of time to best response did not improve the model, but the inclusion of DpR and ETS did. However, the effect of resection cannot be explained by these two variables, because the hazard ratios measuring the effect of resection in both treatment arms remains steady after inclusion in the model of ETS (HR (cet) 0.18, 95% CI 0.04–0.74; HR (bev) 0.83, 95% CI 0.44–1.58; HR (bev) 0.91, 95% CI 0.48–1.73) or DpR (HR (cet) 0.15, 95% CI 0.04–0.63).

## Discussion

In the present analysis, we aimed to identify factors that correlate with resectability and resection in mCRC patients treated in the FIRE-3 study. Resectability was assessed at baseline and at the time of best response by an independent panel based on a central collection of imaging. Resection of metastases was evaluated as actually reported in FIRE-3.

We tried to identify factors that correlate with successful conversion to resectability (not resectable at baseline, but resectable after induction chemotherapy). In addition, using a time-dependent model, we analysed the outcome of resected patients according to the systemic treatment used (cetuximab vs. bevacizumab) within the trial. As these analyses take treatment efficacy into account, the analysis was restricted to patients with *RAS* wild-type tumors. Because primary tumor sidedness did not have a major impact on tumor response,^17^^,^^18^ primary tumor sidedness was not integrated into our analyses—also taking into account that the subgroups would have become even smaller.

Among patients with all *RAS* wild-type tumors, the conversion rate to resectability (according to the central review) was 35% (95/270 patients) with a secondary resection rate of this conversion population of < 12%. By contrast, only one patient that presented with resectable disease at baseline “lost” resectability during the course of therapy and was consecutively not resected. This finding may suggest that in a molecularly favorable population the chance of successful conversion is sufficiently high to stimulate surgical reevaluation in all patients. In addition, the finding that initial chemotherapy may hardly lead to “loss” of resectability could increase the confidence in initial systemic therapy in patients with borderline resectable *RAS* wild-type mCRC.

Among baseline factors, several parameters appeared to be associated with resectability (not successful conversion) and actually performed resection in the trial. However, only age at randomization could be confirmed in a multivariate model. This finding should be interpreted with great care, because the biological background for this finding is unclear and numbers in subgroups might have been limited.

Unlike the pure correlation of parameters and resectability at best response, successful conversion (process of being not resectable at baseline but resectable at best response, according to review) was associated with several significantly negative predictors: presence of *BRAF* mutation, elevated alkaline phosphatase and lung metastasis, which were used to generate a nomogram for the prediction of conversion therapy. Both *BRAF* mutation and elevated alkaline phosphatase are notably unfavourable prognostic factors.^19^^–^21 By contrast, the negative impact of lung metastasis is less clear. In fact, the large database of ARCAD suggests that this (limited) metastatic site does not correlate with unfavorable biology and is rather associated with better outcome than other disease patterns.^22^ A potential explanation could be that pulmonary metastases present in both lungs are less amenable to resection but may be associated with less aggressive dynamics of disease, compared with metastases in other organs, and therefore correlate with favourable survival.

Interestingly, key factors that were positively associated with successful conversion were treatment-associated. In detail, ETS, short time to best response, and DpR were significantly correlated with successful conversion to resectability. This finding is supported by a correlation of response rate and secondary resection of metastases that has been described previously.^4^^,^^23^ This observation speaks to the conclusion that ETS should always stimulate a surgical reevaluation of the patient.

A central aspect of this investigation was the evaluation of survival benefit induced by resection of metastases. Data from prospectively randomized studies regarding this cornerstone of curative therapy are missing, and the estimation of benefit is based on observational cohorts that reflect different eras of drug availability and technical abilities.^1^ The central review of FIRE-3 provides the opportunity to compare outcomes of resected and unresected patients with resectable disease, including a correction for technical difficulty and anticipated clinical benefit. Interestingly, the outcome in resectable and resected patients in this cohort seemed to be influenced by the study arm. This effect was maintained in the fitted Cox model, suggesting that the outcome of resectable and resected patients was more favorable in the cetuximab-arm compared with the bevacizumab-arm of the trial. An obvious explanation for the observed survival differences in the context of resection of metastases is lacking. However, based on the FIRE-3 trial, it might be concluded that cetuximab, as in previous studies, provides a favorable systemic background for the resection of metastases from colorectal cancer.^4^^,^^24^^–^26 It is unclear whether this finding is generalizable to other cohorts with lesions that are considered initially resectable.^27^

Our findings are limited by the lack of a validation cohort with comparable surgical assessment. Furthermore, findings in (sometimes small) subgroups of the presented cohort lack statistical power to demonstrate significant differences.

In conclusion, conversion to resectability in patients with *RAS* wild-type mCRC is significantly associated with baseline characteristics (i.e., lung metastases, *BRAF* mutation, alkaline phosphatase) that also can be combined in a predictive nomogram. Moreover, early efficacy parameters, such as ETS and DpR, correlate with conversion to resectability, suggesting that early response parameters should be meticulously evaluated in the context of potential conversion therapy. In FIRE-3, resection of metastases was associated with improved post-best response survival. This effect originated predominantly from the cetuximab-based study arm, suggesting that EGFR-targeted therapy provides a favorable background for the resection of metastases in mCRC.

## Electronic supplementary material

Below is the link to the electronic supplementary material.

Supplementary material 1 (PDF 133 kb)
