# Supplementary material for: Factors That Influence Conversion to Resectability and Survival After Resection of Metastases in RAS WT Metastatic Colorectal Cancer (mCRC): Analysis of FIRE-3- AIOKRK0306
Source: Ann Surg Oncol. 2020 Mar 14;27(7):2389–401. doi: 10.1245/s10434-020-08219-w (PMC7311511; doi:10.1245/s10434-020-08219-w)
Supplement: Supplementary file 1 — Supplementary material 1 (PDF 133 kb) [file 10434_2020_8219_MOESM1_ESM.pdf]

Suppl Figure 1. Consort Diagram

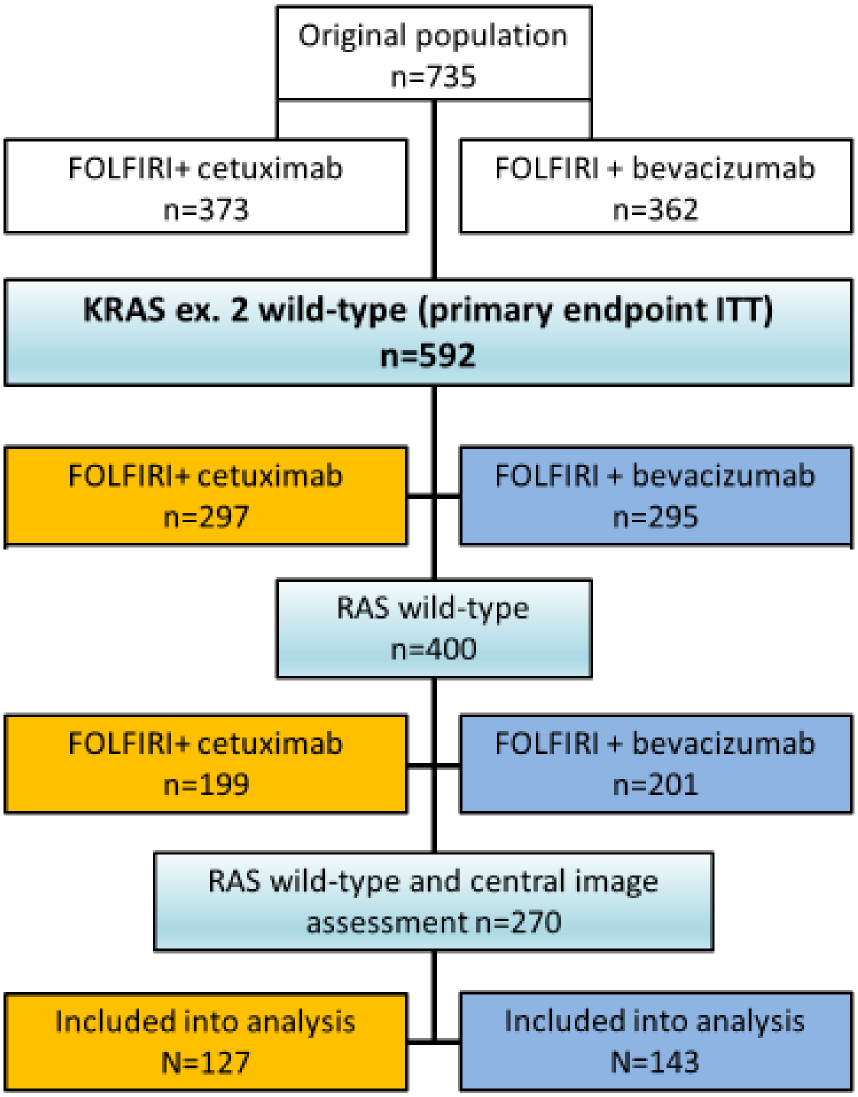

Legend: Illustration of the investigated cohort from the FIRE-3 study.

Suppl Figure 2. Mosaic plots

A. Distribution of the treatment - resectability at best response - resected status

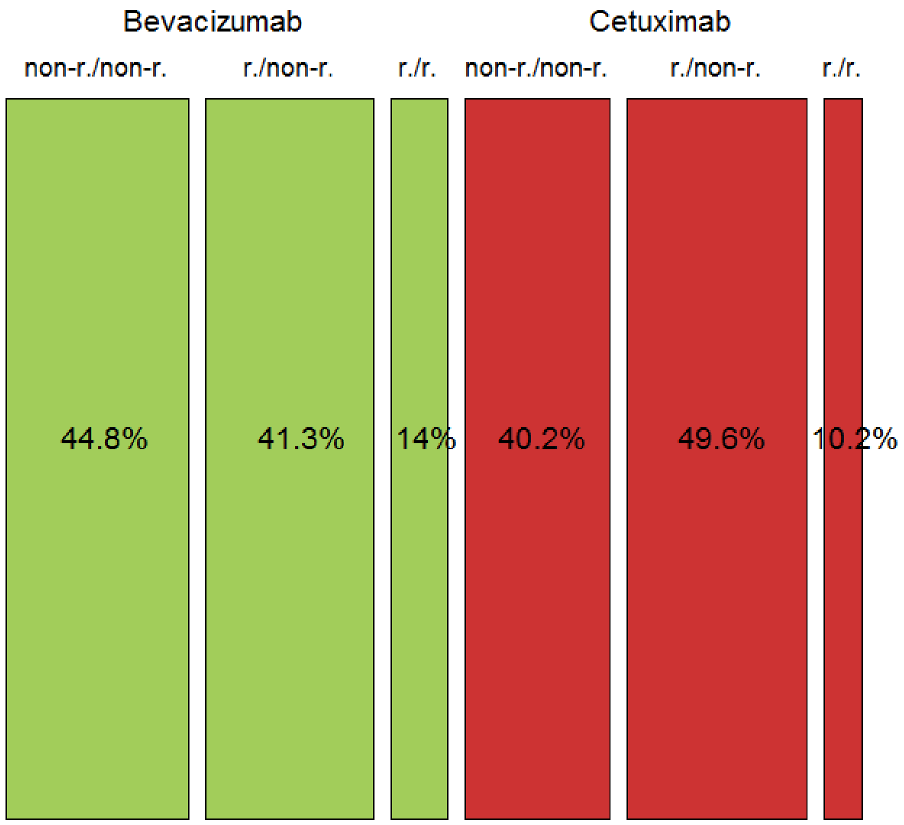

B. Relationship between treatment - resectability at best response - resected status, and presence of lung metastasis at baseline

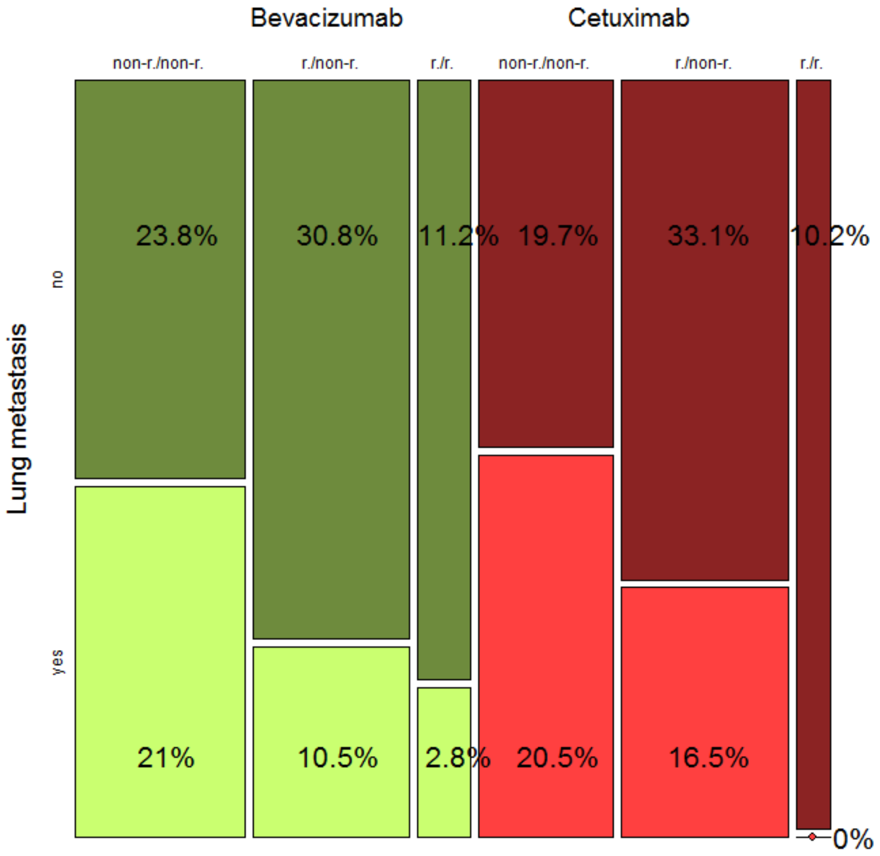

C. Relationship between treatment - resectability at best response - resected status, and presence of metastasis other than lung liver, lymph nodes and peritoneum at baseline

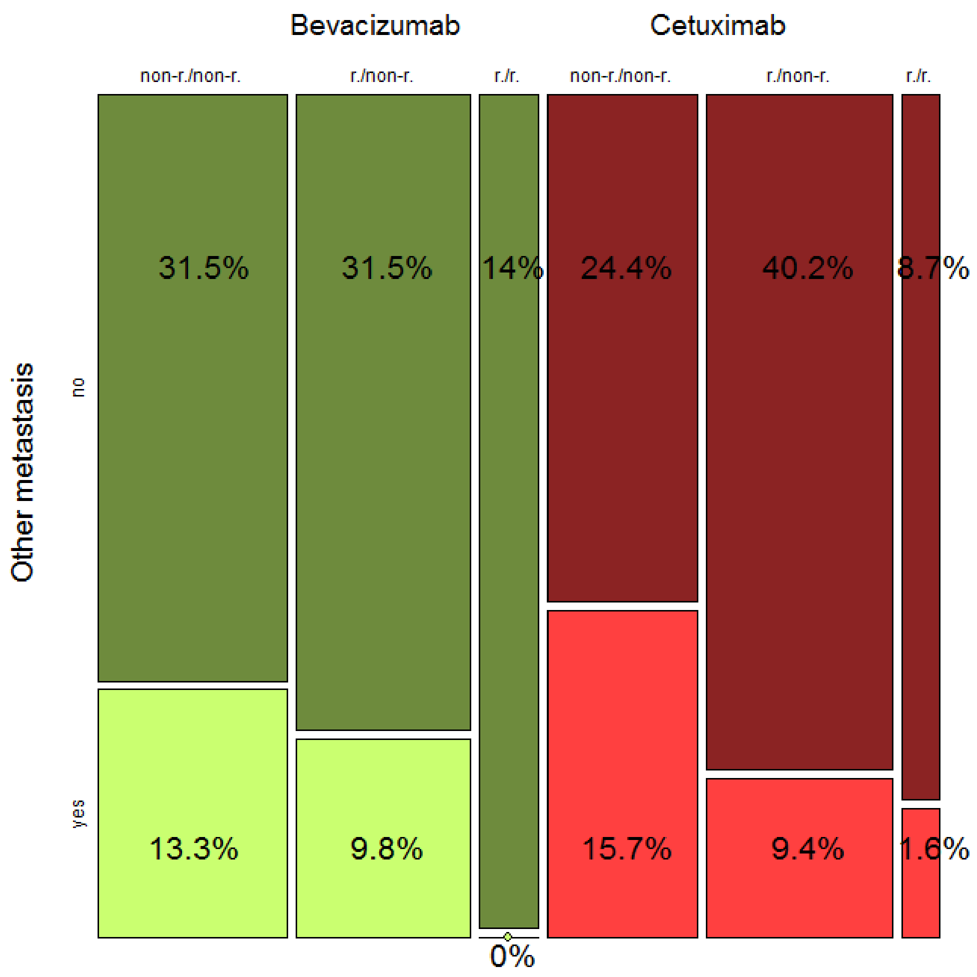

D. Relationship between treatment - resectability at best response - resected status and BRAF mutation

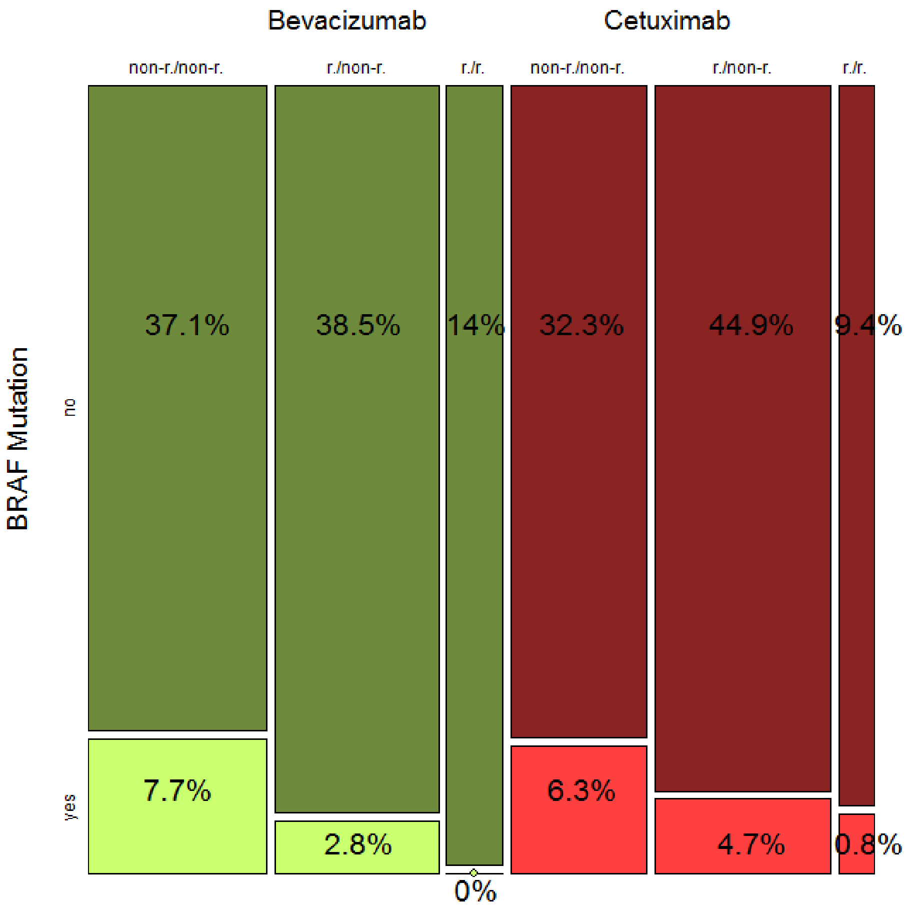

E. Relationship between treatment - resectability at best response - resected status, and alkalic phosphatase level at baseline

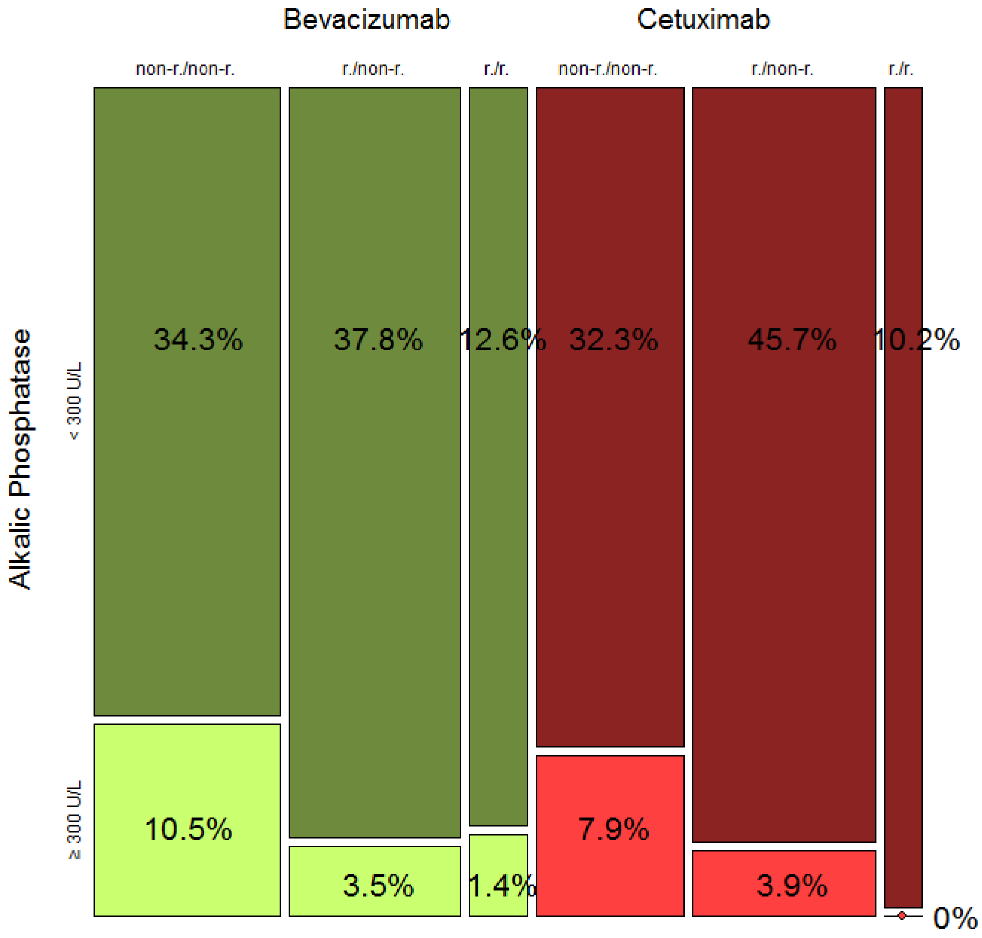

Legend: r: resectable/resected, non-r: not resectable/ not resected. Order: review/reality
